# Effects of Different Extraction Methods on the Structural and Functional Properties of Soluble Dietary Fibre from Sweet Potatoes

**DOI:** 10.3390/foods13152395

**Published:** 2024-07-29

**Authors:** Liuqing Yang, Rongan Zhu, Ning Zhang, Wenya Zhao, Chuyan Wang

**Affiliations:** School of Biology, Food and Environment, Hefei University, Hefei 230601, China; yangliuqing0928@163.com (L.Y.); zhurongan0304@163.com (R.Z.); 17333140632@163.com (N.Z.); zhaowenya0317@163.com (W.Z.)

**Keywords:** sweet potatoes, SDF, structural properties, physicochemical properties, functional properties

## Abstract

In this study, hot water treatment (WT), ultrasonic treatment (UT), ultrasonic-sodium hydroxide treatment (UST), ultrasonic-enzyme treatment (UET), and ultrasonic-microwave treatment (UMT) were used to treat sweet potatoes. The structural, physicochemical, and functional properties of the extracted soluble dietary fibres (SDFs) were named WT-SDF, UT-SDF, UST-SDF, UET-SDF, and UMT-SDF, respectively. Scanning electron microscopy (SEM), Fourier transform infrared spectroscopy (FT-IR), X-ray diffraction (XRD), thermal properties, and Brunauer–Emmett–Teller (BET) analysis were employed. The structural results indicated that the UST-SDF exhibited the best thermal stability, highest crystallinity, and maximum specific surface area. Moreover, compared to hot water extraction, ultrasonic extraction, or ultrasonic extraction in combination with other methods, enhanced the physicochemical and functional properties of the SDF, including extraction yield, water-holding capacity (WHC), oil-holding capacity (OHC), glucose adsorption capacity (GAC), glucose dialysis retardation index (GDRI), sodium cholate adsorption capacity (SCAC), cholesterol adsorption capacity (CAC), nitrite ion adsorption capacity (NIAC), and antioxidant properties. Specifically, the UST-SDF and UMT-SDF showed better extraction yield, WHC, OHC, GAC, CAC, SCAC, and NIAC values than the other samples. In summary, these results indicate that UST and UMT could be applied as ideal extraction methods for sweet potato SDF and that UST-SDF and UMT-SDF show enormous potential for use in the functional food industry.

## 1. Introduction

Sweet potato (*Ipomoea batatas* (L.) Lam) belongs to the *Convolvulaceae* family and is considered vital worldwide. It is rich in nutrients such as starch, protein, phosphorus, calcium, iron, carotene, vitamins, thiamine, riboflavin, and niacin [1]. Sweet potatoes generally contain 8% dietary fibre (DF), and are considered a high-quality food resource with balanced nutrition. Sweet potato resources are abundant; however, deep processing levels are low, mainly focusing on sweet potato starch production. China is the largest producer of sweet potatoes in the world, with fresh sweet potato yields alone exceeding 52 million tonnes in 2019. However, the short storage period and insufficient utilisation have caused a large amount of sweet potato resources to be wasted [2]. Therefore, further effective utilisation of sweet potato resources for deep processing to prepare high-quality DF can maximise resource value.

DF is a widely indigestible nutrient obtained from plant cell walls and is the seventh most important nutrient in living organisms [3]. Dietary fibre is classified into two types based on its capacity to dissolve in water: soluble and insoluble dietary fibre (SDF/IDF) [4]. Compared to IDF, SDF displays better characteristics [5], such as superior solubility, water-/oil-holding capacity, the prevention and treatment of colon cancer, and lower blood cholesterol content [6,7]. SDF is an excellent fat substitute that promotes the flavour of different foods, such as meatballs, dairy products, and baked goods [8,9]. Sweet potatoes are rich in SDF and have high economic value. Therefore, extracting SDF from sweet potatoes could reduce the waste of biological resources.

Recently, SDF extraction methods have included physical, chemical, biological, and composite procedures. Different extraction methods can result in SDFs with different structural, physicochemical, and functional properties [10]. Among these, ultrasound-assisted extraction is widely used to obtain bioactive compounds from various natural sources [11]. Ultrasonic extraction is based on the principle that ultrasound propagates through a liquid medium to induce acoustic cavitation. These cavitation bubbles rupture on the surface of plant materials, generating shock waves and shear forces, thereby damaging plant cell walls and facilitating the further extraction of target analytes [12]. Sodium hydroxide treatment can result in the swelling of plant cell walls and β-elimination of major components, as well as the alteration of cellulose micro-morphology [13]. Microwave treatment can increase intracellular pressure and damage cell walls in fresh tissues, thereby facilitating the release of active ingredients [14]. However, to the best of our knowledge, no study has compared the effects of ultrasound treatment in combination with sodium hydroxide, enzymatic, and microwave methods on the characteristics of SDF from sweet potatoes.

In this study, hot water treatment (WT), ultrasonic treatment (UT), ultrasonic-sodium hydroxide treatment (UST), ultrasonic-enzyme treatment (UET), and ultrasonic-microwave treatment (UMT) were used to extract SDF from sweet potatoes (WT-SDF, UT-SDF, UST-SDF, UET-SDF, and UMT-SDF). The structural, physicochemical, and functional properties of the extracted WT-SDF, UT-SDF, UST-SDF, UET-SDF, and UMT-SDF were compared to screen for a better method for extracting SDF from sweet potatoes.

## 2. Materials and Methods

### 2.1. Materials

Sweet potatoes (watermelon red) were acquired from Sixian (Suzhou, China). These were peeled, cleaned, diced, and dried at 60 °C until the moisture content was below 10%, followed by crushing and screening via a 60-mesh sifter to obtain sweet potato powder. Papain (800,000 U/g) was provided by Beijing Solarbio Science & Technology Co., Ltd., Beijing, China. Cellulase (50 U/g) was provided by Shanghai Yuanye Bio-Technology Co., Ltd., Shanghai, China. Analytical-grade NaOH, 95% ethanol, glucose, cholesterol, sodium gallate, and other chemicals used in the experiments were purchased from Hefei Hongyuan Trading Co., Ltd., Hefei, China.

### 2.2. SDF Extraction

#### 2.2.1. Preparation of WT-SDF

Sweet potato powder (10 g) was added to distilled water (300 mL) and incubated at 90 °C for an hour in an Electro-Thermostatic Water Cabinet (DK-8D, Shanghai Jing Hong Laboratory Instrument Co., Ltd., Shanghai, China). The mixture was continuously stirred at room temperature for 2 h. After centrifugation for 18 min at 4000 rpm, the supernatant was precipitated with four volumes of 95% ethanol for 12 h, and the mixture was then centrifuged at 4000 rpm for 5 min. Finally, the sediment was collected and freeze-dried to obtain WT-SDF.

#### 2.2.2. Preparation of UT-SDF, UST-SDF, UET-SDF, and UMT-SDF

Sweet potato powder (10 g) was added to distilled water (300 mL), and the pH was adjusted to 4.5 using phosphate buffer. Papain (0.6%, *m*/*v*) was added to this mixture and stirred continuously at 60 °C for 40 min, followed by inactivation in a 100 °C water bath for 10 min. Papain-treated samples were collected for subsequent extraction.

This mixture was immersed below the water level in an ultrasonic instrument (L-UCS-15L, LABGIC, Beijing, China) for ultrasonic extraction at an ultrasonic power of 270 W at 40 °C for 30 min. After the treatment described in Section 2.2.1, UT-SDF was obtained. To obtain UST-SDF, the sample was mixed and stirred with a NaOH (5%, *v*/*v*) solution at 40 °C for 30 min under an ultrasonic power of 270 W [15]. The remaining steps were similar to those described in Section 2.2.1. To prepare UET-SDF, the sample was mixed with cellulase (0.6%, *m*/*v*) at 40 °C for 30 min under an ultrasound power of 270 W. The remaining procedures were similar to those described in Section 2.2.1. To obtain UMT-SDF, the sample was stirred at 40 °C for 30 min under an ultrasound power of 270 W and then microwaved for 4 min at 630 W in a microwave oven (P70F20CL-DG(B0), Galanz, Foshan, China). The remaining steps are the same as those presented in Section 2.2.1.

### 2.3. Structural Characterization

#### 2.3.1. Scanning Electron Microscopy (SEM)

The morphology of the SDFs was determined by the method described by Du et al. [16], using a scanning electron microscope (SEM; Regulus 8230, HITACHI, Tokyo, Japan) at 3.0 kV. The SDF samples were placed on double-sided tape and evenly sprayed with a gold layer. Micrographs were captured at 30,000× magnification.

#### 2.3.2. Fourier Transform Infrared Spectroscopy (FT-IR)

The FT-IR spectra of the SDFs were recorded using a Fourier Transform Infrared Spectrometer (Nicolet iS5, Thermo Fisher Scientific, Waltham, MA, USA) within the range of 400–4000 cm^−1^ with a resolution of 4 cm^−1^ and 32 scans. Briefly, the samples (10 mg) were blended with KBr powder (1 g), followed by being ground, and tableted using a tablet press (GS01150, Specac, Kent, UK) at a pressure of 2 tons.

#### 2.3.3. Thermogravimetric Analysis (TGA)

The thermal properties of the SDFs were measured using a thermogravimetric analyser (STA6000, PerkinElmer, Waltham, MA, USA). The sample (20 mg) was heated from 0 °C to 800 °C at a heating rate of 10 °C/min with a flow rate of 20 mL/min under a high-purity N_2_ atmosphere.

#### 2.3.4. X-ray Diffraction (XRD)

The XRD patterns of the SDFs were obtained using an X-ray diffractometer (Ultima IV, Rigaku Corporation, Akishima-shi, Japan). The diffraction angle (2θ) was scanned from 5° to 70° at a scanning rate of 2°/min.

#### 2.3.5. Brunauer–Emmett–Teller (BET) Surface Area

The specific surface areas and pore sizes of the SDFs were measured according to the procedures described by Ma et al. [17] using an analyser (ASAP 2460, Micromeritics, Norcross, GA, USA). Nitrogen adsorption/desorption isotherms were obtained over a relative pressure (P/P_0_) ranging from approximately 10^−5^ to 0.995.

### 2.4. Physicochemical Properties

#### 2.4.1. Colour Analysis

The colour indices of the SDFs, including L* (lightness), b* (redness), and a* (yellowness) values were detected using a colour reader (CR-10, Konica Minolta, Tokyo, Japan).

#### 2.4.2. Water-Holding Capacity (WHC)

The SDF samples (W_1_, 1 g) were added to distilled water (25 mL) at room temperature for 2 h. After 15 min of centrifugation at 4000 rpm by a centrifuge (JW-2032, Anhui Jiawen Instrument Equipment Co., Ltd., Hefei, China), the residue was immediately collected and weighed (W_2_). The WHC was calculated as follows:

WHC (g/g) = (W_2_ − W_1_)/W_1_ (1)

#### 2.4.3. Oil-Holding Capacity (OHC)

The SDF samples (M_1_, 1 g) were added to soybean oil (25 mL) at room temperature for 2 h. Following centrifugation at 4000 rpm for 15 min, the supernatant (free oil) was removed. The residue was weighed (M_2_). The OHC was calculated as follows:

OHC (g/g) = (M_2_ − M_1_)/M_1_ (2)

### 2.5. Functional Properties

#### 2.5.1. Glucose Adsorption Capacity (GAC)

GAC was measured based on Niu et al.’s method with some modifications [18]. The SDF samples (M, 0.1 g) were mixed with 5 mL of a glucose solution (1 mg/mL). The mixture was positioned at room temperature for 2 h and centrifuged at 4000 rpm for 18 min. The concentration of glucose in the supernatant was measured using the DNS method, and GAC was calculated as follows:

GAC (mg/g) = (M_1_ − M_2_)/M (3)

where M_1_ and M_2_ are the weights of glucose in solution before and after adsorption, respectively, and M is the weight of the SDF sample.

#### 2.5.2. Glucose Dialysis Retardation Index (GDRI)

The GDRI was determined in accordance with Qi et al.’s method with some modifications [19]. The SDF samples (0.2 g) were dispersed in 10 mL of glucose solution (1 mM) through a dialysis membrane with a cut-off molecular weight of 1000 D, and incubated in distilled water (200 mL) at 37 °C for 300 min. At 15, 30, 60, 90, 120, 150, 180, 240, and 300 min, 1 mL of this mixture was removed. The DNS method was adopted for measuring the glucose concentration, with the blank group as the control. The GDRI was calculated as follows:

GDRI (%) = (1 − C_1_/C_0_) × 100% (4)

where C_1_ and C_0_ are glucose concentrations in the sample and blank groups, respectively.

#### 2.5.3. Sodium Cholate Adsorption Capacity (SCAC)

SCAC was measured by the method reported by Benitez et al. [20]. The SDF samples (M, 0.2 g) were placed in a 50 mL centrifuge tube with 0.2 g of sodium cholate. The mixture was mixed with 100 mL of NaCl solution (0.15 M), and then incubated at a 37 °C water bath for 3 h. Following 18 min of centrifugation at 4000 rpm, the supernatant (1 mL) was transferred to a glass tube with a plug. Subsequently, the mixture was added to 45% H_2_SO_4_ (6 mL) and 0.3% furfural (1 mL), followed by a 65 °C water bath for 30 min. Finally, the absorbance was measured at 620 nm using a UV-Vis spectrophotometer (UV-1780, Shimadzu Corporation, Suzhou, China) and quantified with the standard curve. SCAC was calculated by the following equation:

SCAC (mg/g) = (M_1_ − M_2_)/M (5)

where M_1_ and M_2_ are the weights of sodium cholate in solution before and after adsorption, respectively, and M is the weight of the SDF sample.

#### 2.5.4. Cholesterol Adsorption Capacity (CAC)

CAC was detected using Gan et al.’s procedure with some modifications [21]. Yolk (10 mL) was mixed with distilled water (90 mL) and stirred to form an emulsion. The SDF samples (M, 0.1 g) were mixed with the emulsion (5 mL), and the pH was adjusted to 7.0. The mixture was shaken at room temperature for 2 h and centrifuged at 4000 rpm for 15 min. The supernatant (1 mL) was collected and diluted in glacial acetic acid (9 mL). Then, the diluent (0.4 mL) was taken to measure the cholesterol concentration at 550 nm and was quantified with the standard curve. An emulsion of diluted eggs without SDF was used as the blank. CAC was calculated by the equation below:

CAC (mg/g) = (M_1_ −M_2_)/M (6)

where M_1_ and M_2_ are the weights of the cholesterol in solution before and after adsorption, respectively, and M is the weight of the SDF sample.

#### 2.5.5. Nitrite Ion Adsorption Capacity (NIAC)

NIAC was evaluated using Zhu et al.’s process [22]. The SDF samples (M, 0.1 g) were added to 5 mL of NaNO_2_ solution (20 μg/mL). The pH was adjusted to 2.0 to imitate the gastric environment. The mixture was incubated at room temperature for 2.5 h and centrifuged at 4500 rpm for 12 min. Subsequently, the supernatant (0.3 mL) was transferred into a tube and deionised water (1.7 mL) was added. Then, 2 mL of p-aminobenzene sulfonic acid (4 μg/mL) together with 1 mL of hydrochloride naphthodiamide (2 μg/mL) was added. After 30 min of reaction in the dark, the concentration of NaNO_2_ was measured at 538 nm and quantified using a standard curve. NIAC was measured through the following equation:

NIAC (μg/g) = (M_1_ − M_2_)/M (7)

where M_1_ and M_2_ are the weights of the NaNO_2_ in solution before and after adsorption, respectively, and M is the weight of the SDF sample.

#### 2.5.6. Antioxidant Properties

Freeze-dried SDF samples (0.2 g) were transferred to test tubes and 50% ethanol (10 mL) was added. This mixture was shaken in a 60 °C water bath for 4 h, and centrifuged at 4500 rpm for 15 min. The supernatants (2 mL) were collected and mixed with ABTS^∙+^ working solution (0.5 mL). The mixture was incubated in the dark for an hour. The ABTS^∙+^ radical scavenging activity was measured by the following equation:

(8) $$\%\;\mathrm{scavenging}\;\mathrm{activity}\;(\%)=\frac{A_{0}-A_{1}}{A_{0}}\times 100\%$$

where A_1_ and A_0_ are the absorbance (734 nm) of the sample and control groups (2.5 mL of ABTS^∙+^ working solution), respectively.

### 2.6. Statistical Analysis

Each test was repeated thrice, and the data were presented as mean ± standard deviation after being statistically processed. SPSS software (version 26.0) was used for *t*-test analysis of intergroup deviation. Differences were considered statistically significant at *p* < 0.05. 

## 3. Results and Discussion

### 3.1. Structural Properties

#### 3.1.1. SEM Observation

**Figure 1:**
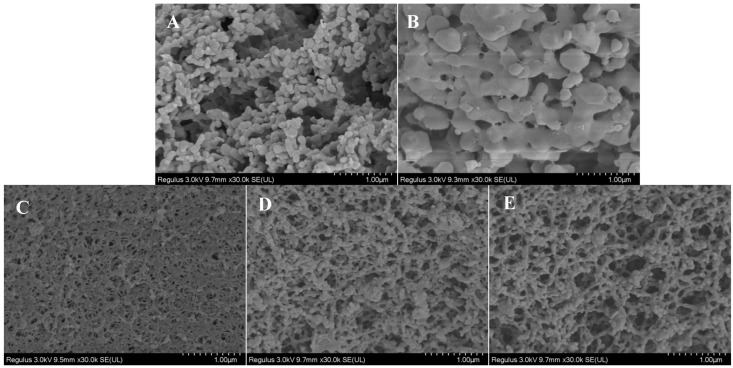
SEM images of WT-SDF (**A**), UT-SDF (**B**), UST-SDF (**C**), UET-SDF (**D**), and UMT-SDF (**E**) with a magnification of 30,000× at 1 µm.

SEM was used to observe the morphological characteristics of the SDF samples (Figure 1). There were significant differences in the morphology of the WT-SDF, UT-SDF, UST-SDF, UET-SDF, and UMT-SDF. The WT-SDF particles aggregated into sheets with smooth surfaces. After UT, the UT-SDF particles connected to form a sheet-like structure with small particles adhering to the surface, and the texture became loose and swollen. Compared to the WT-SDF and UT-SDF, the structures of the UST-SDF, UET-SDF, and UMT-SDF shrank and connected to a network with more unevenly sized pores. The UST-SDF particles became tighter and more disordered, which was probably associated with the strong corrosiveness of NaOH. The UMT-SDF had larger pores and looser textures than the UET-SDF.

#### 3.1.2. FT-IR Analysis

**Figure 2:**
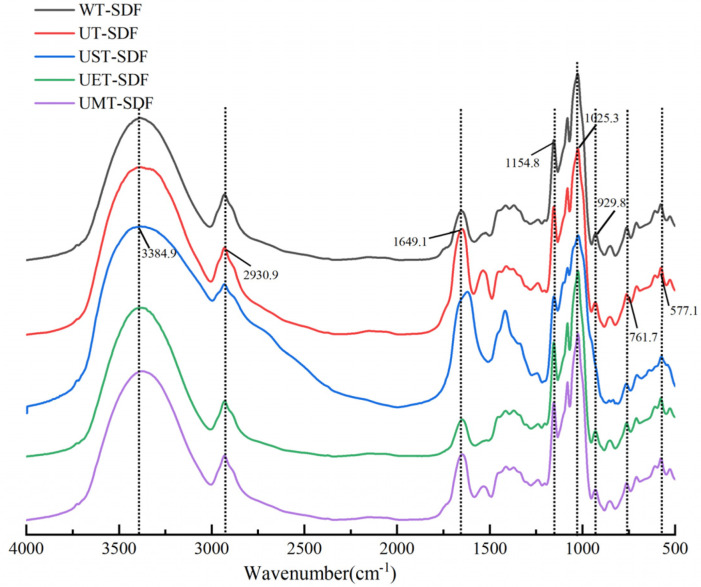
FT-IR spectra of WT-SDF, UT-SDF, UST-SDF, UET-SDF, and UMT-SDF.

The FT-IR spectra of the SDF samples extracted by various methods were recorded within the range from 400 cm^−1^ to 4000 cm^−1^. As shown in Figure 2, several similar characteristic peaks are observed in the spectra of the five SDF samples. The strong and wide peak at 3384 cm^−1^ correlates with the -OH stretching of hydrogen bonds with polysaccharides [18]. Compared with other samples, the UST-SDF exhibited a peak intensity reduction at 2930 cm^−1^, which is a typical absorption peak of polysaccharide compounds, owing to the C-H stretching vibration of the methylene group of the polysaccharides [23,24]. This could probably be caused by the breakage of bonds in the molecule by sodium hydroxide treatment. The absorption peak at 1649 cm^−1^ might be an asymmetric vibration of C=O in the carboxyl group [25]. The peaks at 1200–1400 cm^−1^ were caused by the angular vibration of C-H, which is the characteristic absorption peak of sugars. The spectra of the five SDF samples showed slight differences in this region, especially that of the UST-SDF. The strong absorption peaks at 1154 cm^−1^ and 1025 cm^−1^ are related to the stretching vibration of C-O in C-O-C and the angular vibration of O-H in C-O-H, respectively, which are also characteristic absorption peaks of polysaccharides. Additionally, the peak at 930 cm^−1^ corresponding with the stretching vibration of β-glycosidic linkages was observed in all samples except the UST-SDF sample, demonstrating the degradation of polysaccharide chains caused by sodium hydroxide [26]. The positions of the characteristic absorption peaks in the FT-IR spectra showed no significant changes; however, the intensities of some peaks changed slightly, which may be due to the redistribution of cellulose caused by the different treatment methods.

#### 3.1.3. TGA

**Figure 3:**
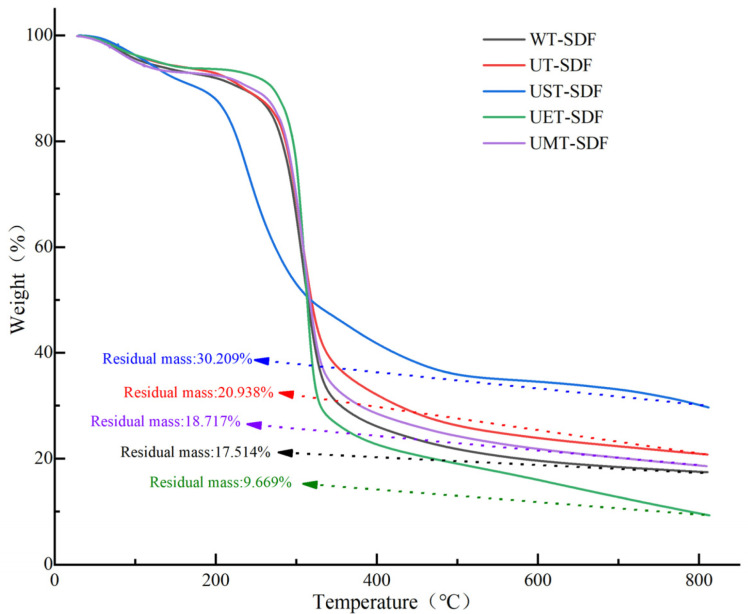
Thermal properties of WT-SDF, UT-SDF, UST-SDF, UET-SDF, and UMT-SDF.

Figure 3 shows the TGA results for the SDF samples. The curves of the five SDF samples exhibited similar trends, and were divided into three temperature ranges. In the first set of temperature ranges (20 °C–200 °C), the curve changed relatively smoothly, and the weight of the sample was decreased slightly, mainly because of the loss of dehydration [27]. In the second set of temperature ranges (200 °C–400 °C), the maximum weight loss was observed, which might be related to the pyrolysis of polysaccharides and hemicellulose [28]. In the final set of temperature ranges (400 °C–800 °C), the weight loss of the SDF samples slowed, mainly due to carbon decomposition. In addition, the residual masses of the WT-SDF, UT-SDF, UST-SDF, UET-SDF, and UMT-SDF were 17.514%, 20.938%, 30.209%, 9.669%, and 18.717%, respectively, indicating better thermal stability of the UST-SDF. Overall, the SDF samples were relatively stable below 200 °C.

#### 3.1.4. XRD Analysis

**Figure 4:**
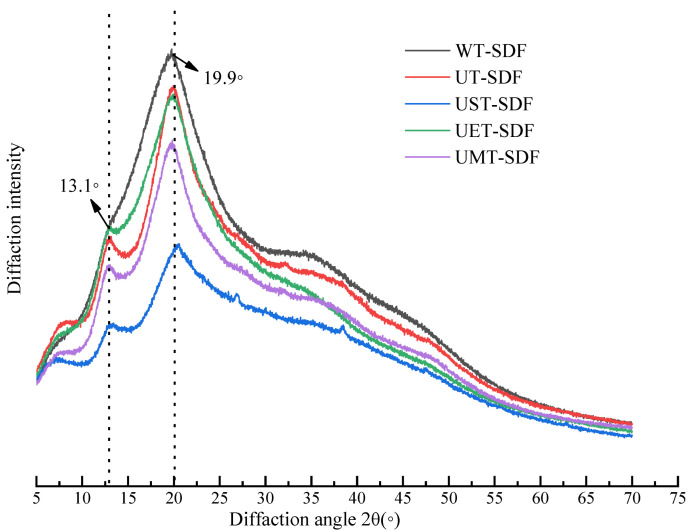
XRD of WT-SDF, UT-SDF, UST-SDF, UET-SDF, and UMT-SD.

XRD was employed to characterise the crystalline morphologies of the polymers. As shown in Figure 4, all the SDF samples had typical crystalline peaks at 13.1° and 19.9°, whereas the prominent diffraction peak was around 19.9°, which is considered a typical structure of cellulose type I. All the SDF samples showed similar XRD patterns, indicating that these extraction methods did not significantly alter the crystal structure of the SDF. Among all the samples, the UST-SDF had the highest crystallinity, followed by the WT-SDF. The crystal structure of SDF is mainly maintained by hydrogen bonds formed between adjacent hydroxyl groups [29]. UT, cellulase hydrolysis, and microwave treatment can result in the degradation of the glycosidic bonds in SDF, resulting in a lower crystallinity of UT-SDF, UET-SDF, and UMT-SDF. The UST-SDF had small diffuse diffraction peaks near 26.9° and 38.3°, as evidenced by the presence of partial crystallisation [8], which is consistent with the UST-SDF having the highest crystallinity of all the SDF samples. This might be due to the combined treatment with ultrasound and alkali, which makes the molecular rearrangement more regular. The UMT-SDF had the lowest crystallinity, suggesting that its crystal structure was severely damaged by the ultrasonic microwaves.

#### 3.1.5. BET Surface Area and Pore Size

**Figure 5:**
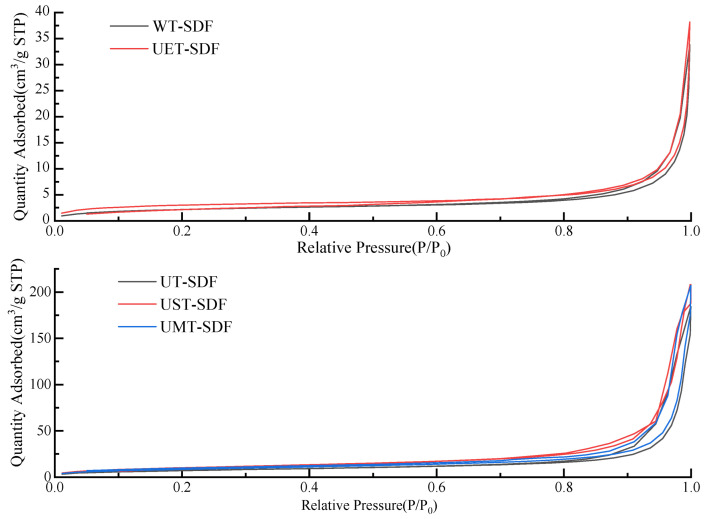
N_2_ Adsorption–desorption of WT-SDF, UT-SDF, UST-SDF, UET-SDF, and UMT-SDF.

The specific surface areas and pore sizes of the SDFs are listed in Table 1. The specific surface areas of the WT-SDF, UT-SDF, UST-SDF, UET-SDF, and UMT-SDF were 7.95, 26.66, 37.86, 10.57, and 31.38 m^2^/g, respectively, with pore sizes of 17.25, 28.33, 26.16, 15.33, and 27.94 nm, respectively. Compared to the WT-SDF, the specific surface area of the other SDF samples increased, and the effect of ultrasonic-assisted NaOH or microwave treatment on the specific surface area increase of the SDF was more obvious than that of UT alone. Moreover, the nitrogen adsorption–desorption isotherms of all the SDF samples were depicted as a V-type isotherm with an H3-type hysteresis loop (Figure 5). In the higher p/p_0_ region, the rate of isotherm ascent increased, and H3-type hysteresis loops were formed, indicating the presence of mesopores and stacking in the SDFs.

### 3.2. Physicochemical Properties

#### 3.2.1. Extraction Yield and Colours of SDFs

The extraction yield and colour parameters (L*, a*, and b*) of the sweet potato SDFs are presented in Table 1. Compared to the WT-SDF, the yields of the UT-SDF (6.27 ± 0.25%), UST-SDF (10.03 ± 0.61%), UET-SDF (7.83 ± 0.12%), and UMT-SDF (8.30 ± 0.30%) were increased, which might be due to the possibility of ultrasound energy increasing the intracellular pressure, rupturing the cell wall and facilitating the further extraction of SDF.

Colour is an essential property that consumers perceive and that often affects their preferences. The L* (white) parameter of the UET-SDF elevated obviously (*p* < 0.05), and the b * (yellow) parameter of the UET-SDF decreased significantly (*p* < 0.05), indicating that the structure of naturally occurring pigments might be degraded under enzymatic conditions. Compared to the other groups, the UST-SDF samples had the lowest L* and a* (red), and the highest b* values, which is likely to be because the sodium hydroxide treatment caused a browning reaction in the SDF [30]. However, the dark colour of the SDF may affect its utilisation in food.

#### 3.2.2. WHC

**Figure 6:**
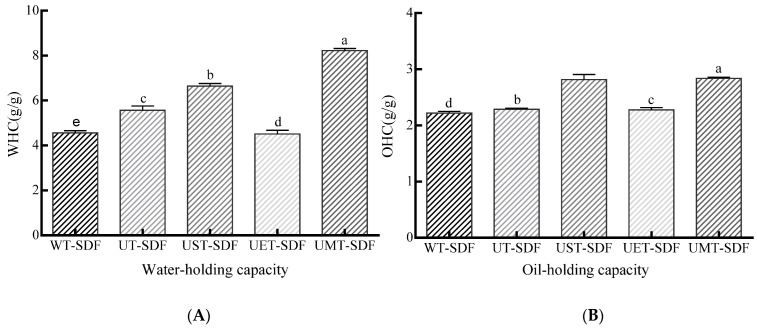
The WHC (**A**) and OHC (**B**) of WT-SDF, UT-SDF, UST-SDF, UET-SDF, and UMT-SDF. Values marked by the different letters (a–e) above are significantly different (*p* < 0.05).

The WHC of the sweet potato SDFs subjected to different treatments is shown in Figure 6A. SDF with a higher WHC prevents food from shrinking and alters its viscosity [31]. As shown in Figure 6A, the UT-SDF, UST-SDF, UET-SDF, and UMT-SDF had higher WHC values than the WT-SDF, indicating that ultrasound alone or in combination with other methods efficiently enhanced the hydration properties of the SDF. The main reason for this is that the porous microstructure and larger specific surface area are both beneficial for the interaction between water and fibres [28]. Compared to the UT-SDF, the WHC of the UET-SDF decreased, which was related to a decrease in the specific surface area and pore size caused by cellulase hydrolysis.

#### 3.2.3. OHC

The OHC of SDF is of great significance for food applications, including avoiding fat loss during cooking [32]. As presented in Figure 6B, the OHC of the WT-SDF, UT-SDF, and UET-SDF displayed little difference and was markedly lower than those of the UMT-SDF (*p* < 0.05) and UST-SDF. The UMT-SDF exhibited the highest OHC, followed by the UST-SDF, although this difference was not of significance (*p* > 0.05), which might be attributed to the exposure of more active groups and an elevation in the specific surface area after the alkaline and microwave treatments.

### 3.3. Functional Properties

#### 3.3.1. GAC

**Figure 7:**
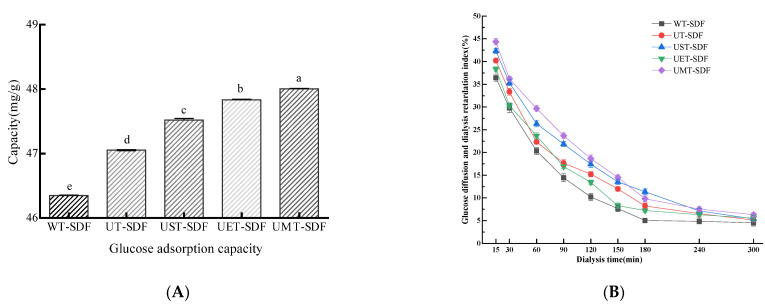
The GAC (**A**) and GDRI (**B**) of WT-SDF, UT-SDF, UST-SDF, UET-SDF, and UMT-SDF. Values marked by the different letters (a–e) above are significantly different (*p* < 0.05).

SDF can combine with glucose in intestinal fluid, thereby reducing postprandial blood glucose levels. GAC is a significant functional property of SDF and is often used to estimate its capacity to postpone glucose absorption. As depicted in Figure 7A, all the SDF samples effectively adsorbed glucose. Compared with hot water extraction, ultrasound treatment effectively enhanced the GAC of the SDF, whereas ultrasound combined with other methods had a better enhancement effect. The UMT-SDF exhibited the strongest adsorption capacity (48.00 ± 0.01 mg/g), which was consistent with the research showing that SDFs with a higher WHC and larger surface area had more of a chance to contact and adsorb glucose [33]. These results suggest that UMT-SDFs exert a strong hypoglycaemic effect in vitro.

#### 3.3.2. GDRI

The GDRI has been used as an effective indicator to evaluate the effects of DFs on delayed glucose absorption in the gastrointestinal tract. As shown in Figure 7B, the inhibitory capacity of the SDF on glucose diffusion decreased continuously with dialysis time, and the final glucose concentration reached equilibrium. The GDRI of the UST-SDF and UMT-SDF was relatively high, possibly due to their increased surface area, which led to the physical hindrance of glucose molecules and stronger interception of glucose in the network structure formed by the fibres [19].

#### 3.3.3. SCAC

**Figure 8:**
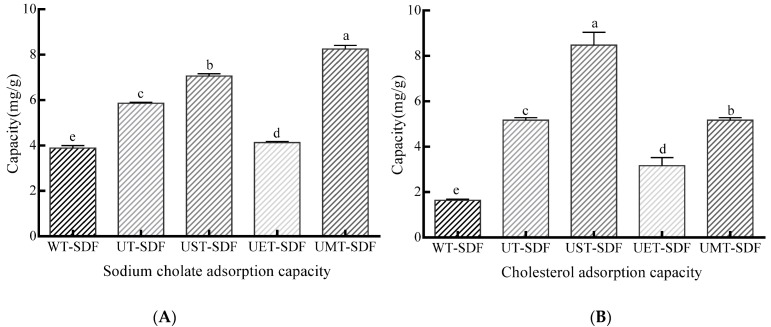
The SCAC (**A**) and CAC (**B**) of WT-SDF, UT-SDF, UST-SDF, UET-SDF, and UMT-SDF. Values marked by the different letters (a–e) above are significantly different (*p* < 0.05).

Sodium cholate is an endogenous emulsifier that plays an important role in lipid digestion and absorption. Therefore, the inhibition of sodium gallate production can effectively reduce fat absorption in the small intestine [34]. Figure 8A shows that the UMT-SDF and UST-SDF had a higher SCAC, which is consistent with their higher OHC, whereas the UST-SDF had a lower SCAC than the UT-SDF. A higher OHC indicates a stronger affinity between the SDF and oil molecules. Therefore, UMT-SDF has a stronger ability to adsorb sodium cholate, effectively reducing the accumulation of bile salts in the circulation in the liver and kidneys.

#### 3.3.4. CAC

CAC is another essential functional property of SDF that reduces serum cholesterol levels and protects cardiovascular function [35]. The CAC of the SDFs is shown in Figure 8B; the WT-SDF had the lowest CAC. Meanwhile, the UMT-SDF and UST-SDF displayed higher CAC values, which might have been caused by their loose structures and larger specific surface areas. Yan et al. [36] found that SDF with more pores could provide more sites for the binding of water and cholesterol, thus possessing a strong CAC. The high SCAC and CAC values of the UMT-SDF and UST-SDF show their potential as ingredients in hypolipidaemic foods.

#### 3.3.5. NIAC

**Figure 9:**
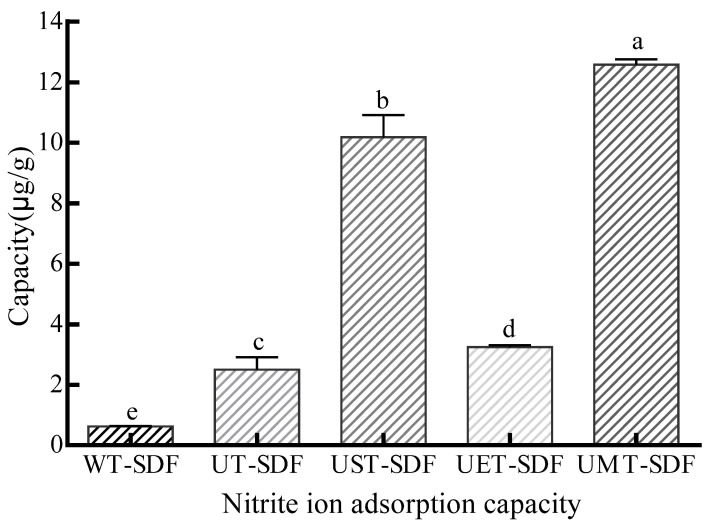
The NIAC of WT-SDF, UT-SDF, UST-SDF, UET-SDF, and UMT-SDF. Values marked by the different letters (a–e) above are significantly different (*p* < 0.05).

Nitrite can react with secondary and tertiary amines/amides to produce nitrosamines with strong carcinogenicity in gastric acid environments. Therefore, the NIAC of SDF is a meaningful functional property. As shown in Figure 9, the adsorption capacity of the SDFs for nitrite ions in a simulated gastric environment was investigated (pH = 2). The results showed that the NIAC of the UMT-SDF was significantly higher than that of the other samples (*p* < 0.05), which might be due to the UMT-SDF having a better structure and a massive explosion of functional groups to interact with more nitrite ions.

#### 3.3.6. Antioxidant Properties

**Figure 10:**
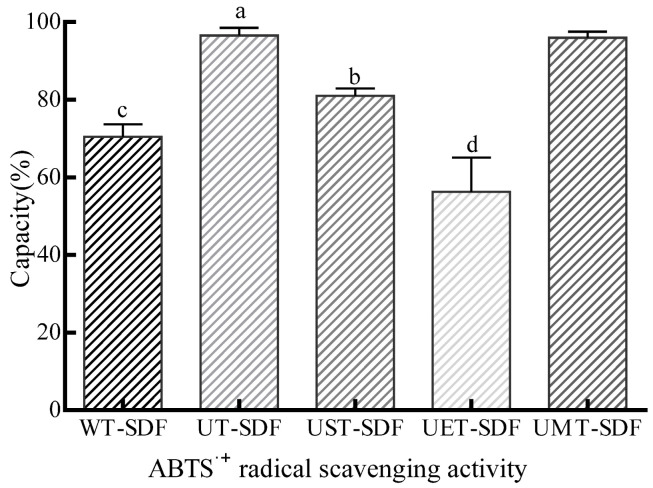
Effects of WT-SDF, UT-SDF, UST-SDF, UET-SDF, and UMT-SDF on radical ABTS^∙+^ scavenging activity. Values marked by the different letters (a–d) above are significantly different (*p* < 0.05).

The ABTS cationic radical model has been widely used to detect T-AOC in SDF [37]. The antioxidant activity was investigated using ABTS^∙+^ radical scavenging activity. Free radical scavenging activities in ABTS^∙+^ are shown in Figure 10. All the samples displayed superior antioxidant activity. The UT-SDF showed the strongest ABTS^∙+^ radical scavenging activity, followed by the UMT-SDF (*p* > 0.05), whereas the UET-SDF showed the lowest activity. Hence, the present study identified sweet potatoes as an important source of antioxidant DF.

## 4. Conclusions

This study employed five methods to extract SDFs from sweet potatoes and investigate their structural, physicochemical, and functional properties. The UST-SDF exhibited the best thermal stability, highest crystallinity, maximum specific surface area, and highest extraction yield. Compared with the WT-SDF and UT-SDF, the UST-SDF and UMT-SDF showed better oil-holding and water-holding, glucose adsorption, sodium cholate, cholesterol adsorption, and nitrite ion absorption capacities, whereas the functions of the UET-SDF were not significantly improved. The UT-SDF had the best antioxidant properties, followed by the UST-SDF and UMT-SDF. Overall, UST-SDF and UMT-SDF have enormous potential for application in the functional food industry.

## Figures and Tables

**Table 1:** Specific surface area, pore size, colours, and extraction yield of WT-SDF, UT-SDF, UST-SDF, UET-SDF, and UMT-SDF.

Sample	Specific Surface Area (m^2^/g)	Pore Size (nm)	L*	a*	b*	Extraction Yield (g/100 g)
WT-SDF	7.95	17.25	84.53 ± 0.46 ^b^	12.27 ± 0.19 ^a^	13.20 ± 0.14 ^b^	3.80 ± 0.20 ^e^
UT-SDF	26.66	28.33	83.23 ± 2.44 ^c^	11.06 ± 0.08 ^b^	11.83 ± 0.34 ^c^	6.27 ± 0.25 ^d^
UST-SDF	37.86	26.16	72.20 ± 1.69 ^e^	4.77 ± 0.05 ^e^	17.83 ± 0.29 ^a^	10.03 ± 0.61 ^a^
UET-SDF	10.57	15.33	88.73 ± 0.95 ^a^	8.93 ± 0.19 ^c^	10.57 ± 0.29 ^e^	7.83 ± 0.12 ^c^
UMT-SDF	31.38	27.94	83.20 ± 0.50 ^d^	5.07 ± 0.12 ^d^	10.67 ± 0.38 ^d^	8.30 ± 0.30 ^b^

Values marked by the different letters (a–e) above are significantly different (*p* < 0.05).

## Data Availability

The original contributions presented in the study are included in the article, further inquiries can be directed to the corresponding author.
